# Combined Application of Organic Fertilizer with Microbial Inoculum Improved Aggregate Formation and Salt Leaching in a Secondary Salinized Soil

**DOI:** 10.3390/plants12162945

**Published:** 2023-08-15

**Authors:** Yuanyuan Peng, He Zhang, Jinshan Lian, Wen Zhang, Guihua Li, Jianfeng Zhang

**Affiliations:** 1 Institute of Agricultural Resources and Regional Planning, Chinese Academy of Agricultural Sciences, 12 Zhongguancun Nandajie Street, Haidian District, Beijing 100080, China; pyysicau@yeah.net (Y.P.); zhanghe@caas.cn (H.Z.); lianjinshan@caas.cn (J.L.); zhangjianfeng@caas.cn (J.Z.); 2 Environment and Plant Protection Institute, Chinese Academy of Tropical Agricultural Sciences, 4 Xueyuan Road, Longhua District, Haikou 570100, China

**Keywords:** secondary salinization, *P. brevitarsis* larvae frass, *B. amyloliticus*, *T. harziensis*, aggregate stability, leaching, plant-growth-promoting microbe

## Abstract

Greenhouse vegetable production provides significant quantities of vegetables throughout the year and improves farmers’ income. However, over-fertilization with mineral fertilizer causes soil secondary salinization and decreases the stability of the soil structure. To improve aggregate formation and decrease salt accumulation in the soil profile, bio-organic fertilizers (*Protaetia brevitarsis* larvae frass with *Bacillus amyloliticus* and/or *Trichoderma harziensis*) were applied to partially substitute mineral fertilizer in a salinized vegetable soil. Soil nutrient condition, aggregate stability, and salt movement in the soil profile were measured in a greenhouse double-cucumber system. The results showed that soil organic matter (SOM), total nitrogen (TN), and available phosphorus (AP) increased significantly under bio-organic fertilizer treatments compared with control. Soil electrical conductivity (EC) and total salt content (TSC) decreased by 15.74–24.20% and 19.15–29.05%, respectively, with bio-organic fertilizers (*p* < 0.05). Cl^−^, NO_3_^−^, and SO_4_^2−^ content under double inoculation with *B. amyloliticus* and *T. harziensis* reduced by 31.19%, 26.30%, and 53.11%, respectively, compared to CK (*p* < 0.05). In addition, double inoculation was more efficient in reducing nitrate content in the soil profile than single inoculation. Soil microaggregates of 0.25–0.053 mm increased by 75.87–78.51% with bio-fertilizers compared with control, and double inoculation was the best for aggregate formation. In conclusion, the inoculation of plant-growth-promoting and salt-tolerant microorganisms with high humic acid larvae frass can alleviate salinization in vegetable soil, enhance soil nutrient content, and improve the soil structure.

## 1. Introduction

The continuous growth in population and the expansion in urbanization increase the competition for crop land. Facility agriculture can effectively alleviate land shortages and promote agricultural production [1,2]. However, in China, the excessive application of mineral fertilizer to achieve high yields, coupled with high temperature and high humidity in greenhouses, has led to serious secondary salinization [3]. As a result, the salt concentration can reach 2.43 g kg^−1^, 141.1% higher than that in the nearby open field [4]. High salt content not only destroys soil aggregates and induces organic matter losses, but also causes an imbalance in soil nutrient condition, thus inhibiting crop growth [5,6].

Therefore, different management methods (such as film mulch, subsurface drainage, and manure and gypsum application) have been used to improve the soil structure and/or reduce salt accumulation. SOM, as a key factor, significantly affects the formation and stability of aggregates, and soil aggregates protect organic matter and reduce its loss [7,8]. Research shows that the combined application of organic and mineral fertilizer can increase the mean weight diameter (MWD) of soil aggregates in coastal saline-alkaline paddy soil [9], and long-term manure application significantly increases the quantity of macroaggregates in sodic soil, particularly water-stable aggregates of 0.25–0.5 mm and 0.5–1 mm [10]. In saline soil, compost (soybean and legumes wastes) application increased macroaggregate formation and aggregate stability by increasing Ca^2+^ content and reducing Na^+^ content [11]. In addition, carbohydrates from sewage sludge increased aggregate stability and decreased the exchangeable sodium percentage [12]. Straw return improved soil aggregate formation and stability in saline soil and affected salt distribution in aggregates [13]. Cattle manure increased organic matter content and exchangeable Ca^2+^ and Mg^2+^ in water-stable aggregates and reduced exchangeable Na^+^ [14]. At the same time, cattle manure can also improve the fractional composition of important substances such as humus compounds [15]. *P. brevitarsis* larvae can decompose lignin- or cellulose-rich organic material, such as mushroom residue or pruned branches from fruit trees, and can directly convert agricultural wastes into organic fertilizer without the need for composting. Frass, as a low-toxicity, humus-rich, and nutrient-rich organic fertilizer, significantly increased tomato and radish yields in a pot experiment, and stimulated the expression of salt-resistant genes in rape [16].

*B. amyloliticus* and *T. harziensis* have been extensively studied as plant growth promotion microorganisms [17]. They can produce or stimulate plants to synthesize various beneficial substances, such as hormones, enzymes, siderophores, and osmosis material, which promote plant growth or maintain plant health in saline conditions [5]. However, their effects on soil structure and hydraulic characteristics have often been neglected. In fact, bacteria or fungi are very important for soil aggregate formation. Bacteria contribute to the formation of both macroaggregates and microaggregates by producing extracellular polymers (sugars, amino acids, and proteins), while fungi enormously influence the polymerization of macroaggregates by mycelial structures in normal soil [17,18,19]. Appropriate *Trichoderma* bio-fertilizer application promoted the formation of macroaggregate (>0.25 mm) and increased soil aggregate stability under non-salt-stressed soil [18]. However, in saline soil, a complicated mixture of 60 microflora with organic materials (total C 13.25%) had no significant impacts on soil organic C content and aggregate formation [19]. Another finding showed that input of soil conditioners with *Bacillus* can influence the formation and stabilization of soil aggregates in saline soil, which is conducive to enhancing salt leaching, reducing surface evaporation and inhibiting salt accumulation in the surface soil [20]. *Trichoderma harzianum* T83, composted with cattle dung, also decreased soil bulk density and soil salt concentration in coastal saline soil [21]. Furthermore, a mixture of three fungi and two bacteria with gypsum can increase saturated hydraulic conductivity of saline-sodic soil [22]. These studies focused on the use of mixed flora with different organic materials, but their results were contradictory. Furthermore, the synergistic effect of *Trichoderma* and *Bacillus* on salinized soil aggregates has not been reported. Therefore, we explored the impact of co-input of organic material with functional microorganisms (salt-tolerant and growth-promoting) on the health of secondary saline soil. We hypothesized that the combined application of *P. brevitarsis* larvae frass with functional microorganisms (*B. amyloliticus* and/or *T. harziensis*) could improve soil fertility and soil structure, enhance salt leaching, and ultimately alleviate secondary salinization. Thus, we measured soil nutrient condition, aggregate formation, and salt movement in the soil profile. 

## 2. Result

### 2.1. Soil Nutrients with Bio-Organic Fertilizer

Bio-organic fertilizer application significantly improved soil properties (Table 1). SOM increased by 11.05%, 11.96%, and 19.16% under T2, T3, and T4 treatments, respectively, compared with CK2. Total nitrogen content of T1, T2, T3, and T4 treatments also significantly increased by 12.75%, 27.25%, 24.95%, and 32.97%, respectively, compared with CK2. Available phosphorus content of T2, T3, and T4 treatments increased by 63.47%, 35.08%, and 27.56%, respectively, compared with CK2. Available potassium under frass treatments (T1–T4) increased 11–15% compared with CK2.

### 2.2. Effect of Bio-Organic Fertilizer on Soil Aggregate Formation

**Figure 1:**
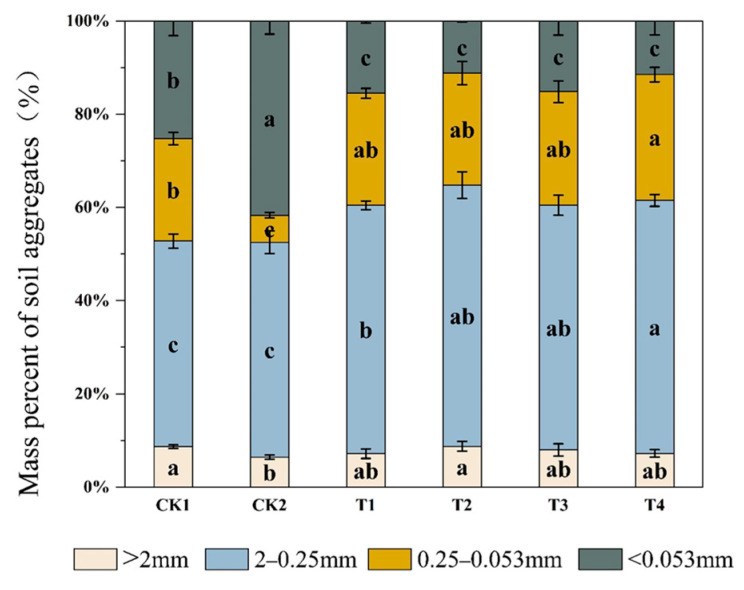
Distribution of water-stable aggregates in the arable layer under different treatments. Full names of the treatment abbreviations can be found in Table 1. Among the different treatments, the same lowercase letters did not differ from each other, *p* ≥ 0.05. The bars stand for mean ± SD.

Bio-organic fertilizer application significantly improved aggregate formation (Figure 1). The proportion of different sizes of aggregates was similar, except for CK2, and was in the order of 2–0.25 mm macroaggregates > 0.25–0.053 mm > smaller than 0.053 mm > larger than 2 mm aggregates. The proportion of macroaggregate and microaggregate under T2 treatment significantly increased by 26.47% and 17.73%, respectively, compared to CK2. In addition, the proportions of microaggregate (0.25–0.053 mm) under T1, T2, T3, and T4 treatments were significantly higher than those of CK2 by 75.89%, 75.87%, 76.18%, and 78.51%, respectively. By comparison, the proportion of silt/clay fraction (<0.053 mm) significantly decreased under T1, T2, T3, and T4 treatments, by 62.80%, 73.16%, 63.53%, and 72.42%, respectively, compared to CK2.

Soil aggregate stability indicators (MWD, GWD, and WR_0.25_) were all greater under bio-organic fertilizer compared with CK2 (Table 2). The MWD under T2, T3, and T4 treatments were significantly higher than those of CK2 by 22.52%, 16.50%, and 14.85%, respectively. The GWD under T2, T3, and T4 treatments was also significantly higher than that of the CK2 by 55.76%, 47.72%, and 51.06%, respectively. Compared to T1 (mineral fertilizer + frass), MWD, GWD, and WR_0.25_ under the T2 treatment were significantly higher, by 10.81%, 17.31%, and 7.69%, respectively.

### 2.3. Effect of Bio-Organic Fertilizer on Soil EC and Total Salt Content

**Figure 2:**
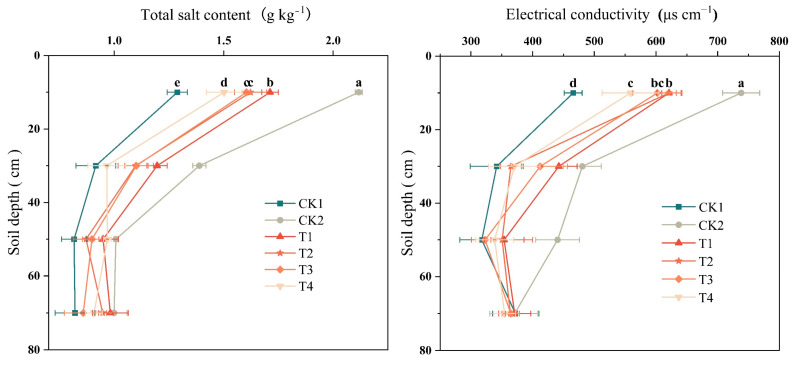
Variation in soil electric conductivity and total salt content under different treatments in different soil profiles. Full names of the treatment abbreviations can be found in Table 1. Among the different treatments, the same lowercase letters did not differ from each other, *p* ≥ 0.05. The bars stand for mean ± SD.

All treatments showed a pattern of gradual decrease in EC and total salt content with increasing soil depth (Figure 2). In the 0–20 cm soil layer, EC significantly reduced by 15.74%, 15.74%, 18.28%, and 24.20% under T1, T2, T3, and T4 treatments, respectively, compared to CK2. The variation pattern of salt content was similar to that of EC, with T1, T2, T3, and T4 treatments reducing by 19.15%, 23.54%, 24.24%, and 29.05%, respectively, compared to CK2. In the 20–40 cm soil layer, soil EC was 26.18% and 24.89% lower with T2 and T4, respectively than CK2. Total salt content was significantly lower with T4 than with CK2. In the 40–60 cm layer, soil EC under bio-fertilizer was all significantly lower with all treatments than with CK2.

### 2.4. Effect of Bio-Organic Fertilizer on Anion and Cation Contents

#### 2.4.1. Soil Anions

**Figure 3:**
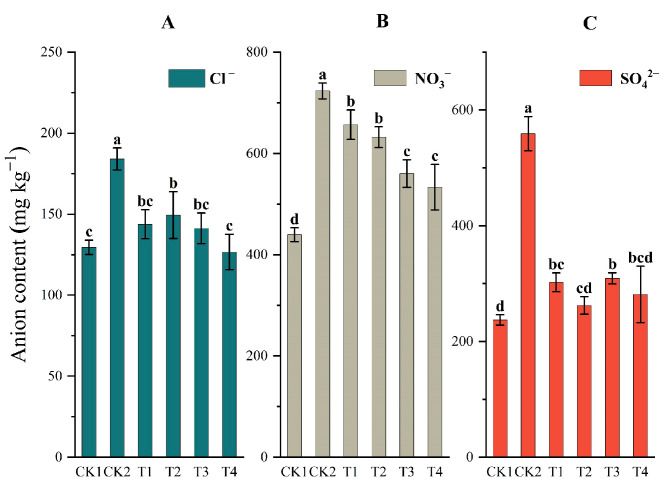
Anion content in the arable layer under different treatments. (**A**) refers to the content of Cl^−^ in different treatments. (**B**) refers to NO_3_^−^ the content of in different treatments. (**C**) refers to SO_4_^2−^ the content of in different treatments. Full names of the treatment abbreviations can be found in Table 1. Among the different treatment, the same lowercase letters did not differ from each other, *p* ≥ 0.05. The bars stand for mean ± SD.

Soil NO_3_^−^ content was much higher than Cl^−^ and SO_4_^2−^ contents in the arable layer under all treatments (Figure 3), and CK2 had the highest content of anions. Compared to CK2 treatment, the Cl^−^ content of T2, T3, and T4 significantly reduced by 18.78%, 23.24%, and 31.19%, respectively (Figure 3A). NO_3_^−^ content also decreased by 9.24%, 12.62%, 22.58%, and 26.30%, respectively (Figure 3B). SO_4_^2−^ content decreased much more (44.73–53.11%) compared with CK2 (Figure 3C).

#### 2.4.2. Soil Cations

**Figure 4:**
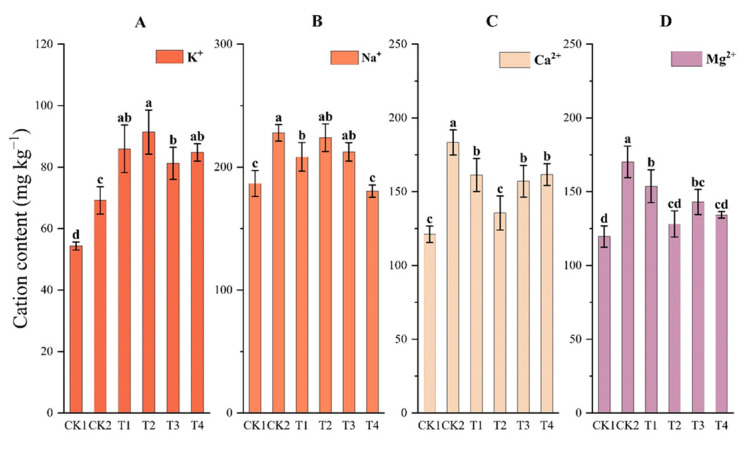
Cation content in the arable layer under different treatments. (**A**) refers to the content of K^+^ in different treatments. (**B**) refers to Na^+^ the content of in different treatments. (**C**) refers to Ca^2+^ the content of in different treatments. (**D**) refers to Mg^2+^ the content of in different treatments. Full names of the treatment abbreviations can be found in Table 1. Among the different treatments, the same lowercase letters did not differ from each other, *p* ≥ 0.05. The bars stand for mean ± SD.

Soil cations were in the order of Na^+^ > Ca^2+^ ≈ Mg^2+^ > K^+^ under all treatments (Figure 4). Bio-organic fertilizer (T2, T3, and T4) significantly increased exchangeable K^+^ content, by 19.49%, 24.28%, and 18.38%, respectively, compared with CK2 (Figure 4A, *p* < 0.05). Soil Na^+^ content decreased 8.59% and 20.78% under T1 and T4, respectively, compared with CK2 (Figure 4B). Soil Ca^2+^ and Mg^2+^ contents decreased 9.69–26.09% under bio-organic fertilizers compared with CK2 (Figure 4C,D, *p* < 0.05). Compared with organic fertilizer treatment (T1), the content of Ca^2+^ and Mg^2+^ under T2 decreased 15.99% and 16.64%, respectively.

### 2.5. Relationships between Soil Aggregates and Soil Salinity, Soil Nutrients

**Figure 5:**
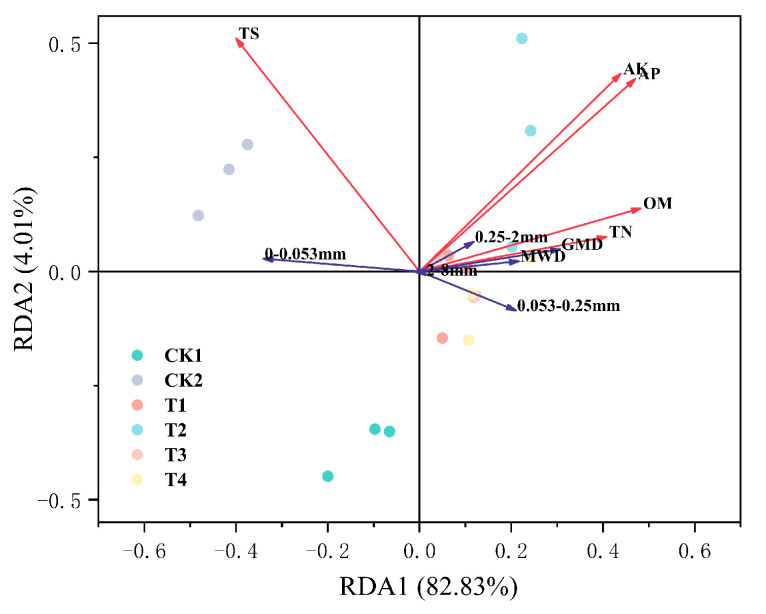
RDA analysis of soil aggregates. Full names of the treatment abbreviations can be found in Table 1. Note: OM means soil organic matter, TN means total nitrogen, AP means available phosphate, AK means available potassium, TS means total salt, and GMD and MWD stand for aggregate stability.

The relationships between soil aggregates and soil properties were analyzed using RDA (Figure 5). The interpretation rate of the first axis is 82.83%. Both soil aggregate (large macroaggregates, macroaggregates, and microaggregates) and aggregate stability (MWD and GWD) were significantly positively correlated with soil nutrients (TN, OM, AP, AK), and significantly negatively correlated with soil total salt content.

### 2.6. Effect of Bio-Organic Fertilizer on Cucumber Yield

**Figure 6:**
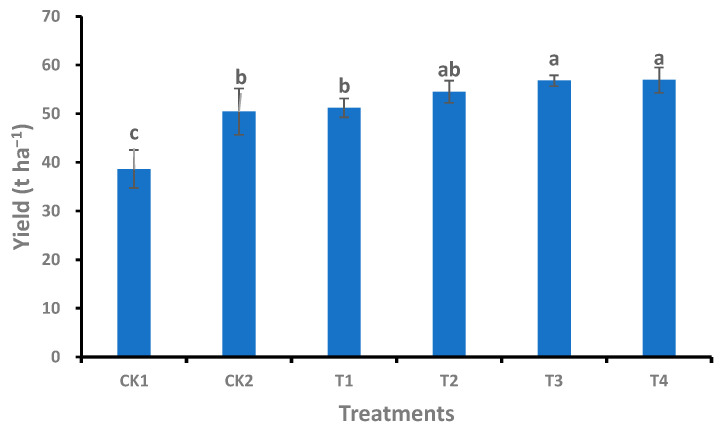
Yield of cucumber under different treatments. Full names of the treatment abbreviations can be found in Table 1. Among the different treatments, the same lowercase letters did not differ from each other, *p* ≥ 0.05. The bars stand for mean ± SD.

Cucumber yield increased by 7.3% and 8.4% with T3 and T4 treatments, respectively, compared to CK2 (Figure 6, *p* < 0.05). Two seasons of no fertilizer application (CK1) led to a significant reduction in cucumber yield compared to CK2. 

## 3. Discussion

Improving the soil structure and increasing infiltration and leaching of salts using organic material input is an efficient way of alleviating secondary salinization in greenhouse soils. We found that the application of *P. brevitarsis* larvae frass (high humic acid) with microorganisms significantly increased SOM. There are two possible reasons: first, the frass directly contributed to SOM; second, the frass provided substrates for microorganisms, and microbial residues or necromass contributed to the organic matter increment [23]. In addition, stable aggregate formation protected organic matter from decomposition [8] and improved salt leaching (Figure 2 and Figure 3). This result was similar to that of Liu et al. [24,25]. They found vermicompost and humic acid enhanced macroaggregate formation and alleviates surface soil salt accumulation. 

Plant-growth-promoting rhizobacteria, such as *B. amyloliticus*, have been extensively studied in saline soil due to their capacity to promote plant growth and disease resistance [26]. However, their “cement” effect on soil aggregates has been neglected. Bacteria inoculation with the frass significantly increased macroaggregate and microaggregate formation and reduced silt/clay content (Figure 1). This can be explained by bacterial secretions (extracellular polysaccharides, proteins, amino acids, etc.) and necromass, which can bond with soil minerals and enhance the formation of stable aggregates [27,28]. In addition, bacteria can form biofilms, which enhance the aggregation effect in a salt-stress environment [29]. Furthermore, soil fungi can influence aggregate formation and the soil structure at different spatial scales through charge, adhesion, and entanglement [27]. This may partly explain why *T. harziensis *with frass promoted aggregate formation (Figure 1). Daynes et al. (2013) also found saprophytic fungi inoculation increased water-stable aggregate formation [30]. However, aggregation disappeared when the fungus died [27]. This may explain why the quantity of macroaggregates inoculated with *B. amyloliticus* was much greater than those inoculated with *T. harziensis*. Meanwhile, double inoculation was more efficient in soil aggregate formation and stability than single inoculation. This is because bacteria and fungi can form synergistic effects, which promote the formation of soil aggregates and improve the stability of the soil structure [31]. 

Application of *P. brevitarsis* larvae frass with microorganisms improved the soil structure, thereby enhancing salt leaching and reducing salt accumulation in the soil profile (Figure 3). This result was consistent with that of Lu and Nisha [32,33]. According to Nisha, applying two *heterocystous cyanobacteria* as bio-fertilizers can improve soil aggregation and structural stability, further significantly decreasing the quantity of sodium ions and electrical conductivity [32]. In addition, *Bacillus* can promote nitrate assimilation [34] and reduce nitrification, thus reducing nitrogen loss [35]. This benefits greenhouse soil in which NO_3_^−^ content is very high.

Na^+^ is one of the main ions that poisons crops and damages the soil structure [5]. We found that frass with double inoculation can significantly decrease Na^+^ (Figure 3). This result is similar to that of Anees et al. (2020) [36]. They found that soil salinity decreased from 6.5 dS m^−1^ to 2 dS m^−1^, and Na^+^ content decreased from 22–24 mmmol L^−1^ to 9–12 mmol L^−1^, when saline soil was inoculated with salt-tolerant bacteria (such as *Bacillus* spp. or *Pseudomonas* spp.). It was speculated that this may be due to extracellular polymeric substances (EPS) produced by salt-tolerant bacteria, promoting the formation and agglomeration of rhizosphere soil [37]. Soil Ca^2+^ and Mg^2+^ are essential elements for crop growth and can exchange mineral Na^+^. However, over-accumulation of the cations hinders crop growth through ion toxicity and impedes the uptake of other beneficial ions [38]. In this study, the cations decreased under *B. amyloliticus* application because of three possible reasons: first, due to leaching; second, due to bridging of Ca^2+^ and Mg^2+^ with EPS produced by *Bacillus*; and, third, due to the participation of Ca^2+^ and Mg^2+^ in aggregate formation [39]. Conversely, in the CK treatment, low soil organic carbon (SOC) content prevented Ca^2+^ from combining with organic matter, ensuring that Ca^2+^ remained free in soil solutions [13].

## 4. Materials and Methods

### 4.1. Site Description

The study site is located in Jiyang District, Jinan City, Shandong Province, China (37°15′ N, 116°52′ E). This area belongs to a sub-humid monsoon climate, with annual mean temperature of 12.8 °C, annual precipitation of 583.3 mm, and annual solar radiation of 124.4 kcal cm^−2^. The soil is coarse sandy loam, developed on the alluvial parent material of the Yellow River. The basic properties of arable soil (0–20 cm) are shown in Table 3.

### 4.2. Experimental Design and Management

The greenhouse has been used for cucumber production for more than 15 years with similar fertilizer management, and the field experiment was carried out in 2022. Cucumbers were repeatedly cultivated twice at the same site under the treatments. Six treatments were designed as follows: (1) CK1: no fertilizer; (2) CK2: 100% mineral N; (3) T1: 70% mineral N + 30% frass N; (4) T2: T1 + *B. amyloliticus*; (5) T3: T1 + *T. harziensis*; (6) T4: T1 + *B. amyloliticus* +*T. harziensis*. Three replicates of each treatment were used in a completely randomized block design with an area of 16 m^2^ (2 m × 8 m) for each plot. Compound fertilizer was applied at the rate of 1386 kg/ha, and contained 208 kg N ha^−1^, 45 kg P ha^−1^, and 86 kg K ha^−1^ as basal fertilizer for CK2. The frass was applied at the rate of 8 t ha^−1^ with 98 kg ha^−1^ mineral N, 45 kg P ha^−1^, and 86 kg K ha^−1^ as basal fertilizer for T1–T4. The application rate of *B. amyloliticus* and *T. harziensis* was 6 L ha^−1^ and 5.4 t ha^−1^, respectively. Mineral fertilizer, frass, and *T. harziensis* (powder) were spread across the soil surface, and *B. amyloliticus* solution (diluted 100 times with water) was sprayed on the soil surface before planting. They were then incorporated into the top 15 cm soil layer. Water-soluble mineral fertilizers were applied five times at the rate of 25 kg N ha^−1^, 6 kg P ha^−1^, and 11 kg K ha^−1^ as topdressing when cucumber began to bear fruits under all treatments except CK1. Six weeks after the start of the experiment, *B. amyloliticus* and *T. harziensis* were re-inoculated as a 100-times diluted solution. 

The *P. brevitarsis* larvae frass (fed on straw and mushroom residue) was provided by Cangzhou Academy of Agricultural and Forestry Science. Basic chemical properties of the frass were 6.07, 58.79%, 1.38%, 1.01%, and 3.26% for soil pH, organic matter, total nitrogen, total phosphorus, and total potassium, respectively. Both *B. amyloliticus* and *T. harziensis* were provided by Agricultural Culture Collection of China (ACCC). The effective number of *B. amyloliticus* was ≥100 million mL^−1^ and the concentration of *T. harziensis* was 0.5 billion g^−1^. Cucumber was grown two seasons per year, irrigated six times in spring and five times in autumn. The irrigation amount, recorded using a water meter, was 0.4 ton per time per plot. Drip irrigation/furrow irrigation was used when the temperature was low/high. Other management practices were in line with those of local farmers.

### 4.3. Soil Sample Collection and Analysis

After cucumbers were harvested in November 2022, soil samples were collected at 20 cm intervals to 80 cm depths. Each sample was air-dried and separated into two parts. One part was passed through a 2 mm sieve and used for soil properties. Total N of arable soil was measured using the Kjeldahl method [40], and available P was extracted with 0.5 M NaHCO_3_ and determined using the method of Olsen et al. [41]. Exchangeable K was extracted with 1.0 M NH_4_OA_C_ (pH 7) and determined using the procedure described by Metson [42]. Soil organic C was determined with the K_2_Cr_2_O_7_ colorimetric oxidization method [43]. SOM was calculated by multiplying the SOC content by the factor 1.724, based on the assumption that SOM contains 58% carbon [44]. Electrical conductivity (EC) and soluble ion content (Na^+^, K^+^, Ca^2+^, Mg^2+^, Cl^−^, SO_4_^2−^, and NO_3_^−^) in different soil layers were measured with soluble extracts (1:5 soil to distilled water) using a Conductivity Meter (conductivity FE30 and electrode LE703, Mettler Toledo, China), and ion chromatography was determined using a conductivity detector (HPIC, 930 Compact IC Flex, Herisau, Switzerland). The cation chromatography column was a Metrosep A supp 4–250/4.0. The anion chromatography column was a Metrosep C 4–100/4.0. The total salt content was obtained by adding seven salt ions (Cl^−^, NO_3_^−^, SO_4_^2−^, K^+^, Na^+^, Ca^2+^, Mg^2+^). 

The other part was passed through an 8 mm sieve and used for aggregate analysis. The aggregate fraction was conducted following Six et al. [8,45]. Firstly, 50 g soil was dry-sieved through a series of three sieves (2 mm, 0.25 mm, and 0.053 mm). Four aggregates fractions were obtained: (i) >2 mm (large macroaggregates), (ii) 0.25–2 mm (macroaggregates), (iii) 0.053–0.25 mm (microaggregates), and (iv) <0.053 mm (silt/clay particles). Second, according to the proportion of dry-sieved matching, 20 g soil was used with a wet sieve. The soil was submerged in deionized water for 5 min and vibrated up and down for 15 min at a rate of 30 times per minute. The fractions remaining on the 2 mm, 0.25 mm, and 0.053 mm sieves were collected, respectively. The silt + clay particles were collected after passing through the 0.053 mm sieve and granular sedimentation. The aggregates were oven-dried (40 °C) and weighed. The index of soil aggregate stability was described by mean weight diameter (MWD) (Equation (1)) [46], geometric mean diameter (GMD) (Equation (2)) [47], and R_0.25_ (Equation (3)). The calculation formulas were as follows:

(1) $$\begin{array}{c} \mathrm{MWD}=\sum_{i=1}^{n}{\bar{x}}_{i}w_{i} \end{array}$$

(2) $$\begin{array}{c} \mathrm{GMD}=\exp\left(\sum_{i=1}^{n}w_{i}\ln{\bar{x}}_{i}\right) \end{array}$$

(3) $$R_{0.25}=\frac{{Mr}_{>0.25}}{M_{T}}\;$$

where *R*_0.25_ represents aggregates larger than 0.25 mm in diameter, M*_T_* represents total mass of the aggregates, and $M_{r}$ represents the mass of aggregates larger than 0.25 mm. ${\bar{x}}_{i}$ is the mean diameter of the aggregate (mm). $w_{i}$ is the weight proportion of each aggregate to the whole soil sample. 

### 4.4. Statistical Analysis

The variance analysis was performed using SPSS software, version 17.0 (SPSS Institute, Inc., Cary, NC, USA). Fisher’s LSD (least significant difference) was used to detect differences between treatments, and the significant differences were determined by LSD at *p* < 0.05. The influence of soil properties on aggregates was analyzed by Redundancy analysis (Canoco 5, Microcomputer Power, Ithaca, NY, USA). All figures were drawn using Origin 2022 (OriginLab, Northampton, MA, USA). All error bars represent the standard deviation. 

## 5. Conclusions

Secondary salinization in greenhouses becomes more serious due to improper management. This study demonstrated that *P. brevitarsis* larvae frass reshaped the soil structure by supplementing organic matter, while inoculation of *B. amyloliticus* and *T. harziensis* further improved aggregation, which promoted salt leaching and reduced soil electrical conductivity and salt content. Overall, the combined application of *P. brevitarsis* larvae frass with plant-growth-promoting *B. amyloliticus* and *T. harziensis* is an efficient means of alleviating soil secondary salinization.

## Figures and Tables

**Table 1:** Soil nutrient content in arable layer in different treatments.

Treatments	SOM	TN	AP	AK
	(g kg^−1^)	(g kg^−1^)	(mg kg^−1^)	(mg kg^−1^)
CK1	57.32 ± 0.71 ^e^	3.04 ± 0.05 ^bc^	227.6 ± 17.15 ^d^	399.1 ± 28.43 ^c^
CK2	59.36 ± 0.70 ^de^	3.03 ± 0.05 ^c^	264.4 ± 13.02 ^c^	472.0 ± 3.41 ^b^
T1	66.32 ± 4.26 ^cd^	3.42 ± 0.87 ^abc^	267.3 ± 14.24 ^c^	522.6 ± 21.26 ^a^
T2	70.74 ± 1.34 ^bc^	3.86 ± 0.30 ^a^	432.2 ± 18.91 ^a^	543.0 ± 7.88 ^a^
T3	74.24 ± 3.16 ^ab^	3.79 ± 0.23 ^ab^	357.2 ± 15.06 ^b^	527.8 ± 20.52 ^a^
T4	78.56 ± 0.55 ^a^	4.03 ± 0.43 ^a^	337.2 ± 16.46 ^b^	536.4 ± 18.63 ^a^

Note: CK1 no fertilizer, CK2 mineral fertilizer, T1 mineral fertilizer + frass, T2 mineral fertilizer + frass + *B. amyloliticus*, T3 mineral fertilizer + frass + *T. harziensis*, T4 mineral fertilizer + frass + *B. amyloliticus* + *T. harziensis.* Values with different lowercase letters in the same column were significantly different at the 0.05 level. Each data point is the mean ± SD. SOM means soil organic matter; TN means total nitrogen; AP means available phosphorus; AK means available potassium.

**Table 2:** Soil aggregate stability index under different treatments.

Treatments	MWD (mm)	GWD (mm)	WR_0.25_ (%)
CK1	0.97 ± 0.03 ^b^	0.32 ± 0.03 ^c^	0.53 ± 0.02 ^c^
CK2	0.86 ± 0.03 ^c^	0.23 ± 0.02 ^d^	0.52 ± 0.02 ^c^
T1	0.99 ± 0.04 ^b^	0.43 ± 0.01 ^b^	0.60 ± 0.08 ^b^
T2	1.11 ± 0.04 ^a^	0.52 ± 0.01 ^a^	0.65 ± 0.02 ^a^
T3	1.03 ± 0.04 ^b^	0.44 ± 0.02 ^b^	0.60 ± 0.01 ^b^
T4	1.01 ± 0.04 ^b^	0.47 ± 0.04 ^b^	0.61 ± 0.01 ^b^

Note: The abbreviations for mean weight diameter, geometric mean diameter, and >0.25 mm water- stability aggregate are MWD, GWD, and WR_0.25._ Values with different lowercase letters in the same column were significantly different at the 0.05 level. Full names of the treatment abbreviations can be found in Table 1. Each data point is the mean ± SD.

**Table 3:** Soil properties before field experiments.

pH	SOM (g kg^−1^)	TN (g kg^−1^)	AP (mg kg^−1^)	AK (mg kg^−1^)	TSC (g kg^−1^)	EC (dS m^−1^)
7.26	62.5	3.23	289.9	137.5	4.63	1.1

Note: SOM means soil organic matter; TN means total nitrogen; AP means available phosphorus; AK means available potassium; TSC means total salt content.

## Data Availability

Not applicable.
